# A Novel Prediction Model of Acute Kidney Injury Based on Combined Blood Variables in STEMI

**DOI:** 10.1016/j.jacasi.2021.07.013

**Published:** 2021-10-26

**Authors:** Yuhei Goriki, Atsushi Tanaka, Kensaku Nishihira, Nehiro Kuriyama, Yoshisato Shibata, Koichi Node

**Affiliations:** a Department of Cardiovascular Medicine, National Hospital Organization Ureshino Medical Center, Saga, Japan; b Department of Cardiovascular Medicine, Saga University, Saga, Japan; c Miyazaki Medical Association Hospital Cardiovascular Center, Miyazaki, Japan

**Keywords:** acute kidney injury, biomarker, myocardial infarction, AKI, acute kidney injury, AMI, acute myocardial infarction, AUC, area under the curve, BS, blood sugar, eGFR, estimated glomerular filtration rate, hsTnI, high-sensitivity troponin I, IABP, intra-aortic balloon pumping, PCI, percutaneous coronary intervention, STEMI, ST-segment elevation myocardial infarction

## Abstract

**Background:**

Development of acute kidney injury (AKI) is associated with poor prognosis in patients with ST-segment elevation myocardial infarction (STEMI).

**Objective:**

This study sought to investigate whether a combination of pre-procedural blood tests could predict the incidence of AKI in patients with STEMI.

**Methods:**

A total of 908 consecutive Japanese patients with STEMI who underwent primary percutaneous coronary intervention within 48 hours of symptom onset were recruited and divided into derivation (n = 617) and validation (n = 291) cohorts. A risk score model was created based on a combination of parameters assessed on routine blood tests on admission.

**Results:**

In the derivation cohort, multivariate analysis showed that the following 4 variables were significantly associated with AKI: blood sugar ≥200 mg/dL (odds ratio [OR]: 2.07), high-sensitivity troponin I >1.6 ng/mL (upper limit of normal ×50) (OR: 2.43), albumin ≤3.5 mg/dL (OR: 2.85), and estimated glomerular filtration rate <45 mL/min/1.73 m^2^ (OR: 2.64). Zero to 4 points were given according to the number of those factors. Incremental risk scores were significantly associated with a higher incidence of AKI in both cohorts (*P* < 0.001). Receiver-operating characteristic curve analysis of risk models showed adequate discrimination between patients with and without AKI (derivation cohort, area under the curve: 0.754; 95% confidence interval: 0.733-0.846; validation cohort, area under the curve: 0.754; 95% confidence interval: 0.644-0.839).

**Conclusions:**

Our novel laboratory-based model might be useful for early prediction of the post-procedural risk of AKI in patients with STEMI.

Acute kidney injury (AKI) is a frequent complication in patients with coronary artery disease undergoing coronary intervention, and it is known that patients with ST-segment elevation myocardial infarction (STEMI) have a higher risk of AKI (1,2). The incidence of AKI in patients with STEMI is approximately 10% to 20% (3,4), and the occurrence of AKI critically affects in-hospital and long-term outcomes (5,6). The mechanisms underlying AKI in the clinical setting of STEMI and subsequent primary percutaneous coronary intervention (PCI) are multifactorial (7). Contrast agents are known to have direct cytotoxic effects on renal tubular cells, as well as indirect cytotoxic effects through changes in renal blood flow causing regional hypoxia (8,9). In contrast, key pathogenetic mechanisms of AKI result from systemic and renal hemodynamic changes secondary to impaired cardiac output and increased venous congestion (4). Moreover, an imbalance of endogenous vasodilatory and vasoconstrictive factors is involved (10). A burst of excess immunological and inflammatory activation is also likely the potential cause of further renal injury (5). Thus, these pathophysiological responses that occur during the post–acute myocardial infarction (AMI) phase have a strong association with the occurrence of AKI.

Several risk score models have been developed to assess the incidence of AKI, including the Mehran score (11, 12, 13, 14). Mehran’s model requires 8 variables to assess the risk of AKI, as follows: age >75 years, hypotension, congestive heart failure, hemoglobin, estimated glomerular filtration rate (eGFR), diabetes, contrast volume, and need for intra-aortic balloon pumping (IABP). However, the volume of contrast material administered and use of a hemodynamic support device are unknown before the procedure.

To date, several blood biomarkers have been reported to be potential tools for predicting AKI in patients with AMI (13,15, 16, 17, 18). Given that these biomarkers may reflect different aspects of pathophysiological responses that occur during the post-AMI phase, we hypothesized that a combination of biomarkers might provide more accurate and useful information for risk stratification than the information provided by any individual biomarker. In this study, we sought to develop a risk score prediction model, based on a combination of parameters obtained on routine blood tests, for in-hospital AKI in patients with STEMI who underwent primary PCI and to compare the predictive utility of that model with that of the conventional Mehran model.

## Methods

### Study design and population

**Figure 1:**
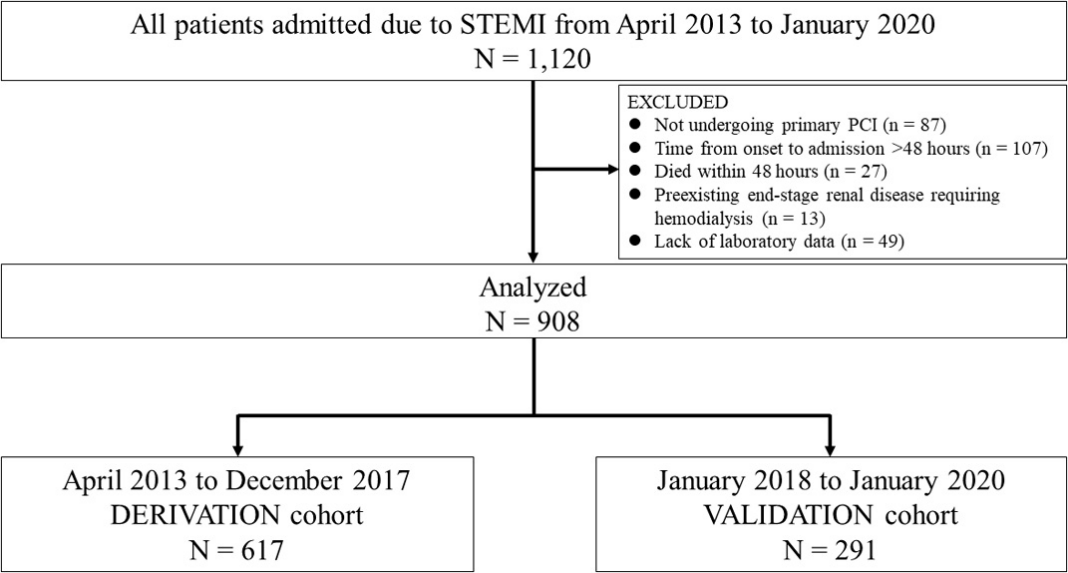
Patient Sample Selection PCI = percutaneous coronary intervention; STEMI = ST-segment elevation myocardial infarction.

This was a single-center, retrospective observational study undertaken in Japan. A total of 1,120 consecutive patients who were admitted to Miyazaki Medical Association Hospital for STEMI between April 2013 and January 2020 were enrolled in the study. Exclusion criteria were the following: 1) not receiving primary PCI; 2) time from onset to admission >48 hours; 3) died within 48 hours; 4) pre-existing end-stage renal disease requiring dialysis; and 5) lack of laboratory data on admission. A total of 908 patients were ultimately included in the study. Based on the index date of hospital admission, they were divided into 2 groups, the derivation and validation sets, which consisted of 617 patients hospitalized from April 2013 to December 2017 and 291 patients hospitalized from January 2018 to January 2020, respectively (Figure 1). The study protocol was approved by the Institutional Review Board at Miyazaki Medical Association Hospital (2021-2).

### Diagnosis of STEMI

STEMI was diagnosed according to the universal definition of myocardial infarction put forth by the European Society of Cardiology and the American Heart Association (19). After urgent admission to our hospital and within 48 hours after onset of symptoms, all patients suspected to have STEMI according to the clinical manifestations, such as electrocardiogram changes and elevated cardiac enzymes, received emergency coronary angiography and subsequent coronary revascularization. Reperfusion therapy was performed along with primary PCI according to the relevant guidelines and recommendations (20). Then, the patients received optimal medications (20).

### Definition of AKI

The initial serum creatinine level at admission was used to determine baseline renal function. The definition of AKI was based on change in serum creatinine using the Kidney Disease: Improving Global Outcomes criteria, as follows (21): stage 1, a rise in serum creatinine by ≥0.3 mg/dL within 48 hours, or an increase in serum creatinine of at least 1.5 times the baseline that is known or presumed to have occurred within the prior 7 days; stage 2, more than a 2- to 2.9-fold increase in serum creatinine; and stage 3, more than a 3-fold increase in serum creatinine from baseline or initiation of renal replacement therapy or an increase in serum creatinine to >4.0 mg/dL.

### Data collection

The following types of data were collected: demographic characteristics, medical history, presenting signs and symptoms, results of blood tests, transthoracic echocardiographic and electrocardiographic findings, cardiac procedures, and in-hospital outcomes. The white blood cell count, platelet count, and levels of hemoglobin, C-reactive protein, creatinine, creatine kinase, blood sugar (BS), glycosylated hemoglobin, uric acid, low-density lipoprotein cholesterol, high-density lipoprotein cholesterol, albumin, high-sensitivity troponin I (hsTnI), and B-type natriuretic peptide were measured in blood specimens obtained immediately after admission. The eGFR was calculated using the revised equation for the Japanese population (22).

### Statistical analysis

Continuous variables are reported as mean ± SD for normally distributed values and as median (interquartile range) for non-normal values. Categorical variables are expressed as number and percentage. Comparisons of continuous variables between groups were performed with the Student *t* test or Mann-Whitney *U* test, as appropriate. Comparisons of categorical variables were assessed with the chi-square or Fisher’s exact test, as appropriate. To develop the predictive model, we divided patients into 2 groups based on the index date of hospital admission to form the derivation and validation sets. As a sensitivity analysis, we also randomized these patients 7:3 to either the derivation or validation cohort with statistical software and performed the same analysis in this randomized derivation cohort. To develop the prediction model, we categorized the blood variables significantly associated (*P* < 0.05) with the incidence of AKI in univariate analysis and retained them for multivariate analysis. Continuous variables were categorized using clinically useful cutoff values (13, 14, 15, 16, 17, 18). These potential risk markers were eliminated by multivariate logistic regression analysis using backward factor elimination (*P* < 0.05), and the variables selected were given an equal weight of 1 point to develop a risk score model. The patients were then classified into 3 groups according to the total risk score, as follows: low risk (0 or 1 point), moderate risk (2 points), and high risk (3 or 4 points). Statistical trends among groups were evaluated with the Cochran-Armitage trend test. Discrimination of the risk score for predicting AKI was assessed using receiver operating characteristic curve analysis. Calibration was assessed using the Hosmer-Lemeshow goodness-of-fit test and was satisfied when the *P* value was >0.05. We further calculated the AUC of Mehran’s model (11) and compared it with that of the risk score. The analyses were performed with the JMP software program, version 14.2.0 (SAS Institute). Values of *P* < 0.05 were considered statistically significant.

## Results

### Patient characteristics

A total of 908 patients (617 in the derivation cohort and 291 in the validation cohort) were analyzed. Table 1 shows the patients’ admission characteristics as stratified by study cohort. No significant differences were seen between the derivation and validation cohorts for clinical parameters, vital signs, and laboratory data, with the exception of high-density lipoprotein cholesterol. No significant differences in clinical parameters related to treatments for STEMI were observed between the 2 cohorts. In-hospital deaths occurred in 4.5% of patients in the derivation cohort and in 2.8% of patients in the validation cohort.

**Table 1:** Comparisons of Baseline Characteristics Between the Derivation and the Validation Cohorts

	Derivation Cohort (n = 617)	Validation Cohort (n = 291)	*P* Value
Age, y	68.7 ± 12.8	68.9 ± 12.1	0.818
Male	458 (74.2)	208 (73.4)	0.381
Body mass index, kg/m^2^	23.9 ± 3.7	24.0 ± 3.8	0.592
Systolic blood pressure, mm Hg	138.2 ± 32.5	135.2 ± 33.2	0.088
Heart rate, beats/min	77.1 ± 20.6	74.9 ± 22.0	0.145
Medical history			
Hypertension	423 (68.6)	189 (65.0)	0.251
Dyslipidemia	337 (54.6)	149 (51.2)	0.332
Diabetes mellitus	185 (30.0)	88 (30.2)	0.937
Smoking	322 (52.2)	149 (51.2)	0.781
Previous MI	64 (10.3)	33 (11.3)	0.659
Previous PCI	66 (10.7)	29 (10.0)	0.734
Previous CABG	6 (1.0)	3 (1.0)	0.938
Laboratory date			
WBC, ×10^2^/μL	106.7 ± 38.0	104.4 ± 37.3	0.472
Hemoglobin, g/dL	14.0 ± 2.0	14.0 ± 2.1	0.982
Platelet, ×10^4^/μL	22.4 ± 6.2	22.1 ± 6.0	0.496
HbA1c, %	6.0 (5.7-6.5)	6.0 (5.7-6.5)	0.604
BS, mg/dL	157 (133-202)	161 (132-204)	0.803
eGFR, mL/min/1.73 m^2^	67.7 ± 22.1	65.0 ± 20.4	0.101
LDL-CHO, mg/dL	126.3 ± 35.0	125.4 ± 36.2	0.784
HDL-CHO, mg/dL	46.2 ± 11.8	48.5 ± 13.3	0.012
Uric acid, mg/dL	5.9 ± 1.6	5.8 ± 1.5	0.521
CRP, mg/dL	0.13 (0.06-0.40)	0.13 (0.06-0.35)	0.371
CK, IU/L	153 (94-396)	176 (94-429)	0.384
hsTnI, ng/mL	0.27 (0.04-2.42)	0.20 (0.03-2.32)	0.324
BNP, pg/mL	41.2 (17.2-141.1)	52.7 (16.9-148.2)	0.579
Killip classification			
Ⅰ/Ⅱ/Ⅲ/Ⅳ	505/63/20/29	237/21/9/24	0.195
Onset to admission time, min	210 (120-417)	195 (112-390)	0.520
LVEF (on admission), %	50.4 ± 10.6	49.0 ± 9.9	0.054
Culprit lesion			
LMT	14 (2.3)	4 (1.4)	0.122
LAD	312 (50.6)	164 (56.3)	0.223
RCA	228 (37.0)	97 (33.3)	0.311
LCX	63 (10.2)	26 (8.9)	0.249
Multivessel disease	224 (36.3)	88 (30.2)	0.072
Pre-TIMI flow grade 0.1	406 (65.8)	194 (66.7)	0.797
Post-TIMI flow grade 3	584 (94.6)	265 (91.0)	0.081
Peak CK level, IU/L	2,131 (1,099-3,931)	2,234 (1,090-3,817)	0.909
Mechanical support on admission			
Respirator	27 (4.4)	16 (5.5)	0.460
Temporary pacing	48 (7.8)	34 (11.8)	0.056
IABP	80 (13.0)	38 (13.1)	0.969
ECMO	18 (2.9)	7 (2.4)	0.660
In-hospital death	28 (4.5)	8 (2.8)	0.197
AKI	49 (7.9)	28 (9.6)	0.497
Stage Ⅰ	39 (6.3)	22 (7.6)	0.437
Stage Ⅱ	5 (0.8)	2 (0.7)	0.553
Stage Ⅲ	5 (0.8)	4 (1.3)	0.254

Values are mean ± SD, n (%), median (interquartile range), or n.

AKI = acute kidney injury; BNP = B-type natriuretic peptide; BS = blood sugar; CABG = coronary artery bypass grafting; CK = creatine kinase; CRP = C-reactive protein; ECMO = extracorporeal membrane oxygenation; eGFR = estimated glomerular filtration rate; HbA1c = glycosylated hemoglobin; HDL-CHO = high-density lipoprotein cholesterol; HR = heart rate; hsTnI = high-sensitivity troponin I; IABP = intra-aortic balloon pumping; LAD = left anterior descending; LCX = left circumflex; LMT = left main trunk; LDL-CHO = low-density lipoprotein cholesterol; LVEF = left ventricular ejection fraction; MI = myocardial infarction; PCI = percutaneous coronary intervention; RCA = right coronary artery; TIMI = Thrombolysis In Myocardial Infarction; WBC = white blood cell.

In the derivation and validation cohorts, 7.9% and 9.6% of patients, respectively, had AKI (any stage). Of these, 6.3% and 7.6%, respectively, had AKI stage 1; 0.8% and 0.7%, respectively, were AKI stage 2; and 0.8% and 1.3%, respectively, were AKI stage 3.

### Estimation of risk factors for AKI

Table 2 shows the univariate analysis of the results of blood testing in the derivation cohort, stratified by AKI or non-AKI. The variables that were significant by univariate analysis were subjected to multivariate stepwise backward logistic regression analysis (Table 3), and 4 variables were ultimately proved to be significantly associated with AKI, as follows: BS ≥200 mg/dL, hsTnI >1.6 ng/mL, albumin ≤3.5 mg/dL, and eGFR <45 mL/min/1.73 m^2^. Zero to 4 points were given according to the number of factors a patient had.

**Table 2:** Univariate Analysis of Variables for In-Hospital AKI in the Derivation Cohort

	Non-AKI	AKI	*P* Value
WBC, ×10^2^/μL	104.5 ± 36.2	113.1 ± 43.7	0.027
Hemoglobin, g/dL	14.1 ± 2.0	13.5 ± 2.4	0.027
Platelet, ×10^4^/μL	22.6 ± 6.2	20.3 ± 6.1	0.018
HbA1c, %	6.0 (5.7-6.5)	6.0 (5.6-7.3)	0.532
BS, mg/dL	156 (132-195)	191 (149-282)	<0.001
eGFR, mL/min/1.73 m^2^	68.7 ± 21.5	56.1 ± 26.3	<0.001
LDL-CHO, mg/dL	126.2 ± 34.6	126.7 ± 38.9	0.937
HDL-CHO, mg/dL	46.6 ± 11.9	42.9 ± 9.9	0.036
Albumin, mg/dL	4.1 ± 0.4	3.7 ± 0.6	<0.001
Uric acid, mg/dL	5.9 ± 1.5	6.5 ± 1.7	0.004
CRP, mg/dL	0.12 (0.06-0.37)	0.25 (0.05-0.74)	0.010
CK, U/L	147 (93-363)	267 (130-1,232)	0.002
hsTnI, ng/mL	0.22 (0.03-1.97)	1.82 (0.23-19.7)	<0.001
BNP, pg/mL	37.7 (17.0-122.0)	139.1 (27.0-373.0)	<0.001

Values are mean ± SD or median (interquartile range).

Abbreviations as in Table 1.

**Table 3:** Multivariate Logistic Regression Analysis in the Derivation Cohort and Corresponding Risk Score for AKI

	Odds Ratio	95% Confidence Interval	*P* Value	Score
BS ≥200 mg/dL	2.07	1.08-3.94	0.026	1
hsTnI >1.6 ng/dL (normal upper limit×50)	2.43	1.30-4.55	0.005	1
Albumin ≤3.5 mg/dL	2.85	1.41-5.79	0.004	1
eGFR <45 mL/min/1.73 m^2^	2.64	1.32-5.28	0.006	1

Abbreviations as in Table 1.

### Sensitivity analysis

In a sensitivity analysis, the same 4 variables as those selected in a derivation cohort based on the index date were significantly associated with the incidence of AKI (Supplemental Tables 1 and 2).

### Laboratory-based prediction of AKI

**Figure 2:**
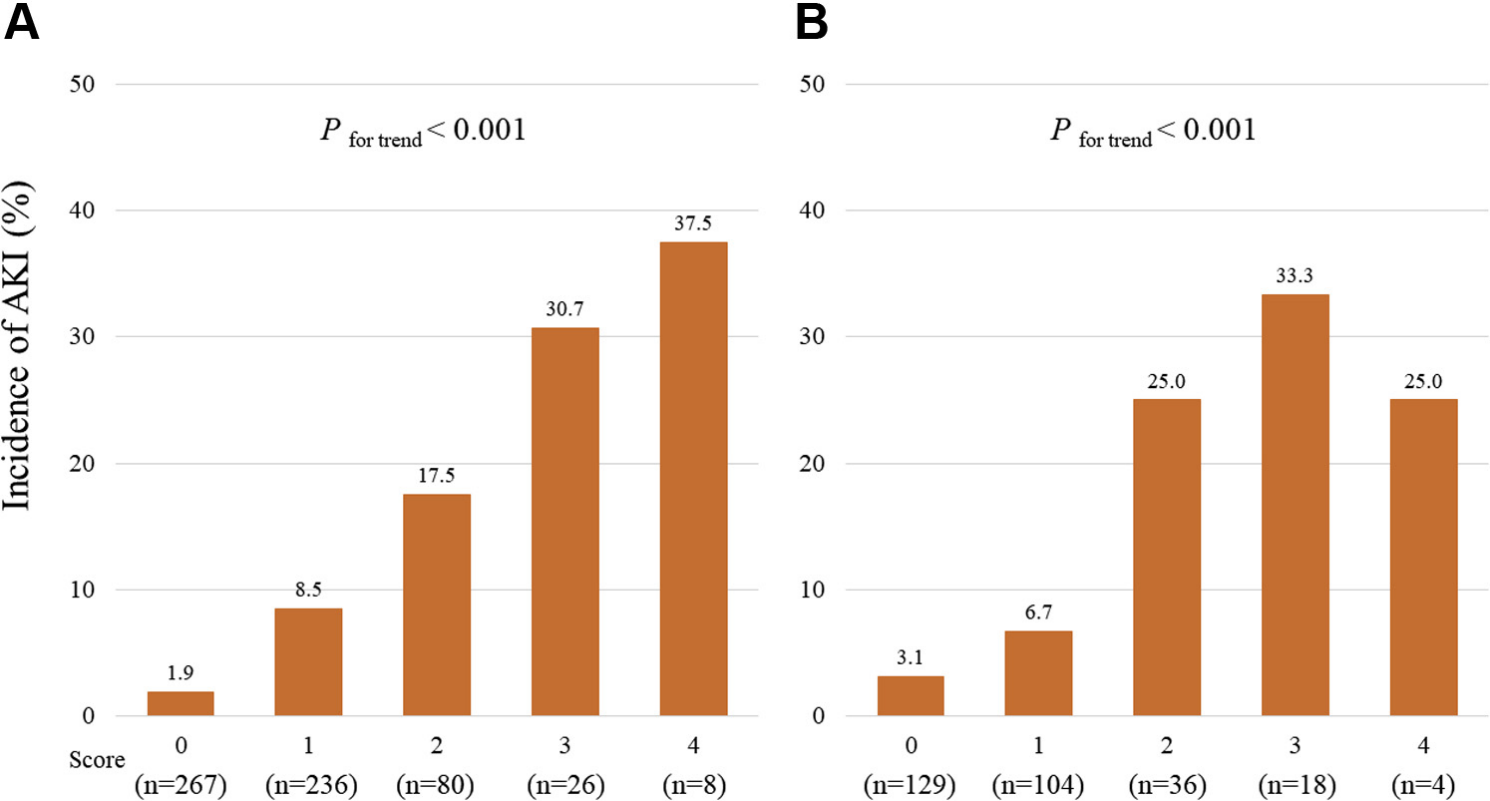
Incidence of In-Hospital AKI According to the Laboratory-Based Scores **(A)** Derivation and **(B)** validation cohorts. AKI = acute kidney injury.

**Figure 3:**
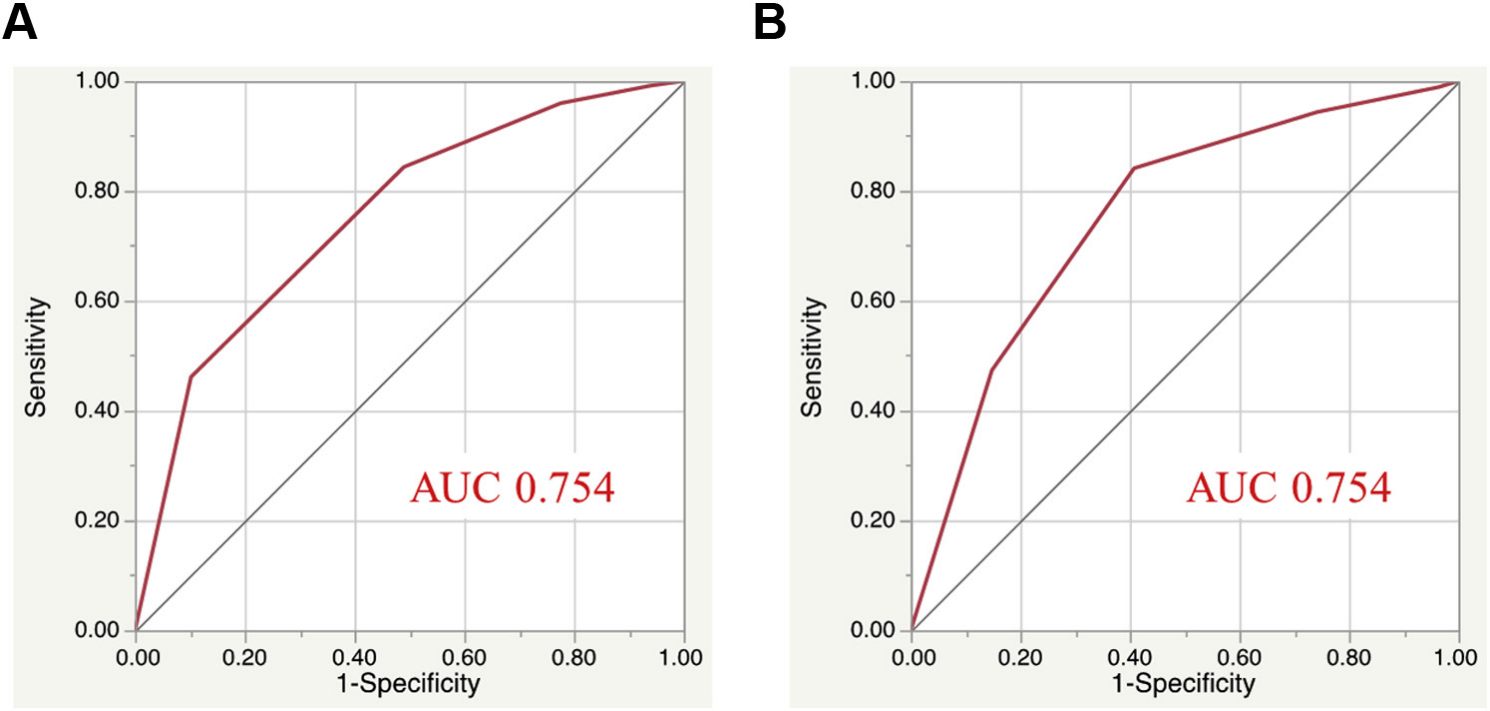
Receiver Operating Characteristic Curves of Laboratory Risk Score **(A)** Area under the curve (AUC) was 0.754 (95% confidence interval: 0.682-0.814) for the derivation cohort. **(B)** AUC was 0.754 (95% confidence interval: 0.644-0.839) for the validation cohort.

**Figure 4:**
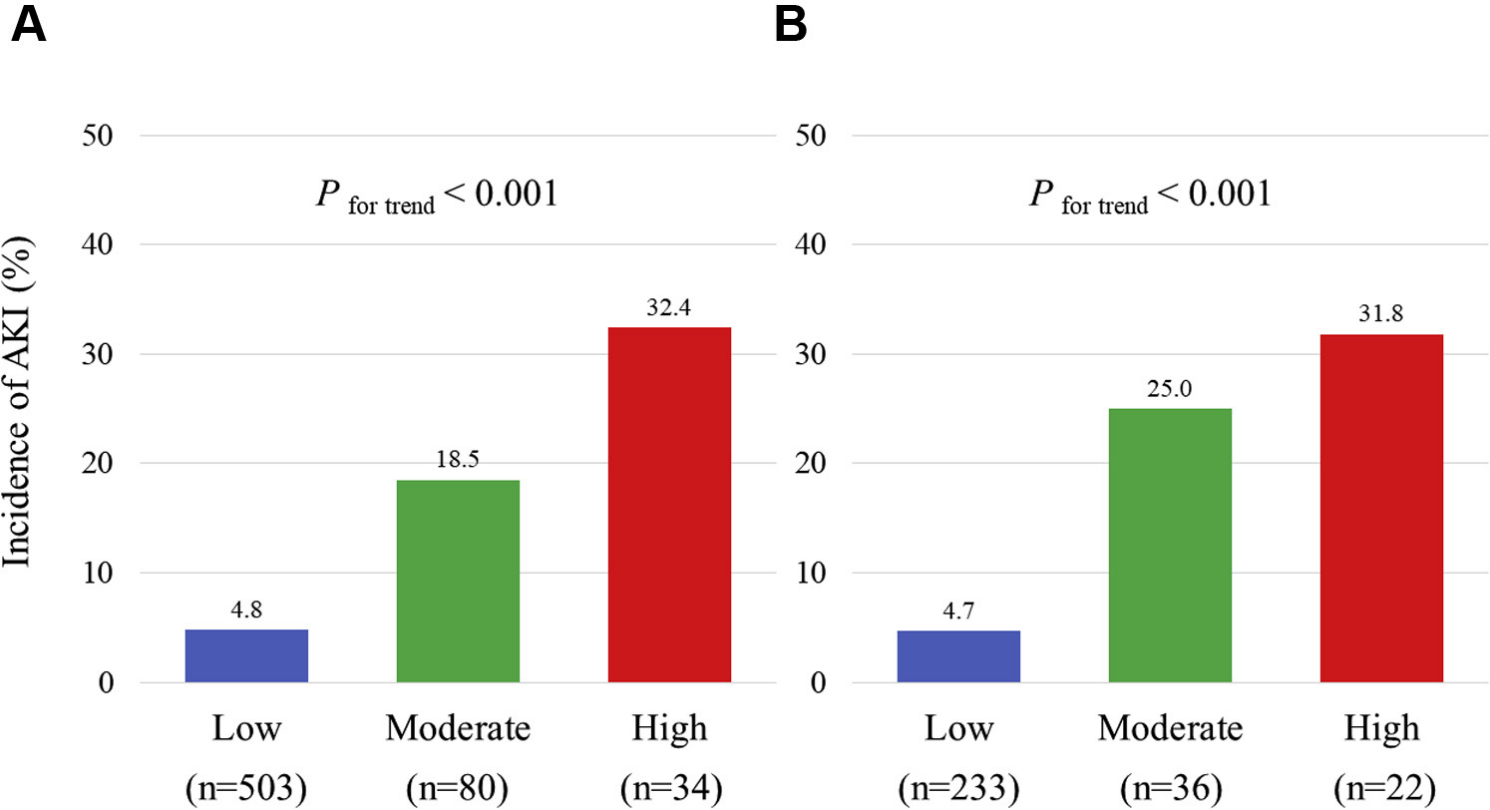
Incidence of In-Hospital AKI in Subgroups Stratified by the Laboratory-Based Risk Model **(A)** Derivation and **(B)** validation cohorts. The scores of the low-, moderate-, and high-risk subgroups are defined as 0 or 1, 2, and 3 or 4, respectively. AKI = acute kidney injury.

In the derivation cohort, an increased total risk score was significantly associated with an elevated incidence of AKI (*P*_for trend_ < 0.001) (Figure 2A). The risk score also showed a significant trend for the incidence of AKI in the validation cohort (*P*_for trend_ <0.001) (Figure 2B). The risk model displayed adequate discrimination between patients with or without AKI in the validation (Figure 3A) and derivation (Figure 3B) cohorts. The Hosmer–Lemeshow statistic suggested a good fit in both the derivation cohort (chi-square = 1.87, *P* = 0.600) and the validation cohort (chi-square = 1.61, *P* = 0.870). Additionally, we classified the patients into 3 groups according to risk score to simplify its use in clinical settings: a low-risk group (0 or 1 point), a moderate-risk group (2 points), and a high-risk group (3 or 4 points). These subgroups also showed a significant trend for in-hospital incidence of AKI among the respective validation and derivation cohorts (Figures 4A and 4B).

### Comparison with Mehran’s model

**Figure 5:**
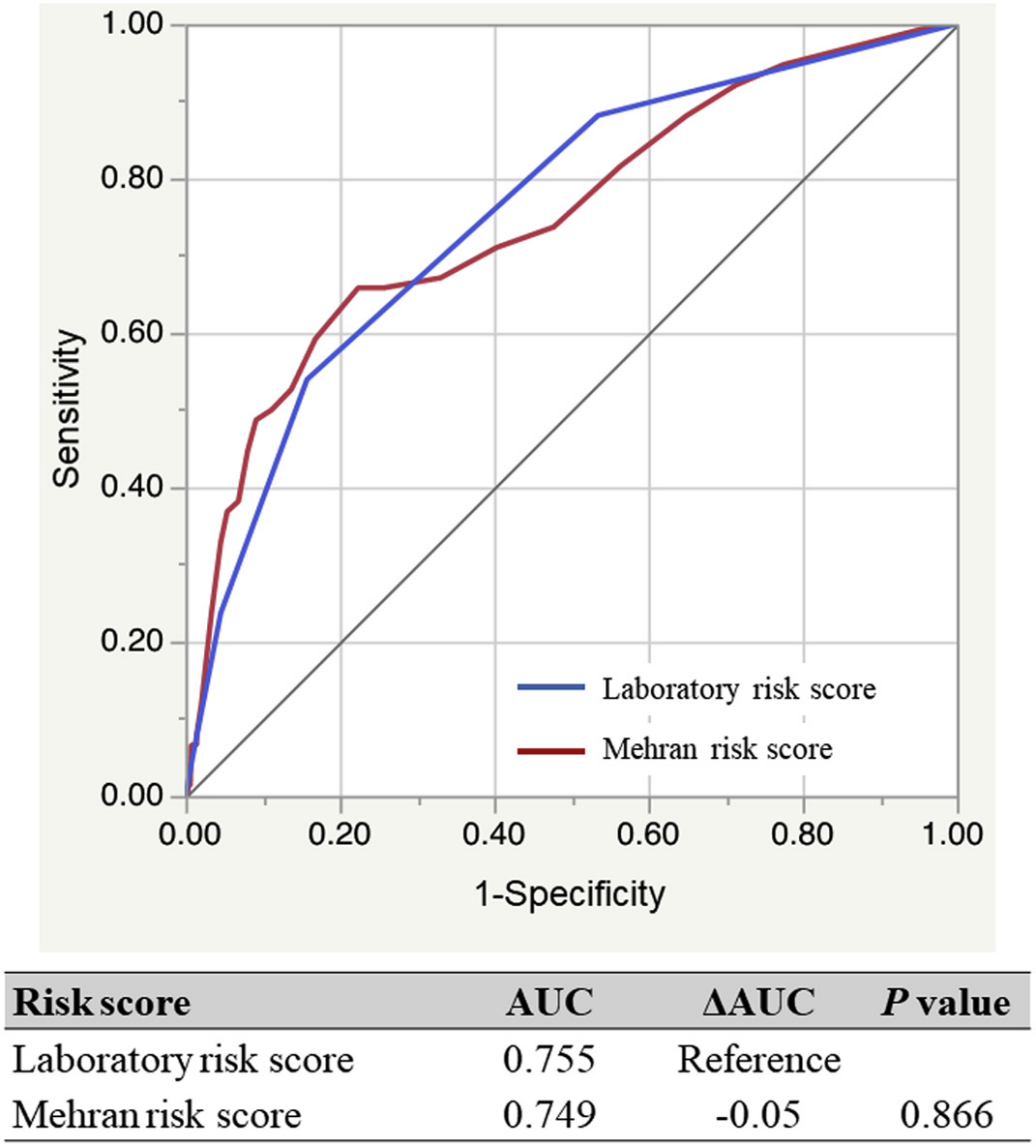
Comparison of Predictive Values for In-Hospital AKI between the Laboratory-Based Model and the Mehran Model Areas under the curves (AUCs) of the laboratory-based model **(blue)** and the Mehran model **(red)** in all cohorts were 0.755 (95% confidence interval: 0.696-0.805) and 0.749 (95% confidence interval: 0.681-0.807), respectively, and the difference in predictive ability between models was not significant (*P* = 0.866). AKI = acute kidney injury.

**Figure 6:**
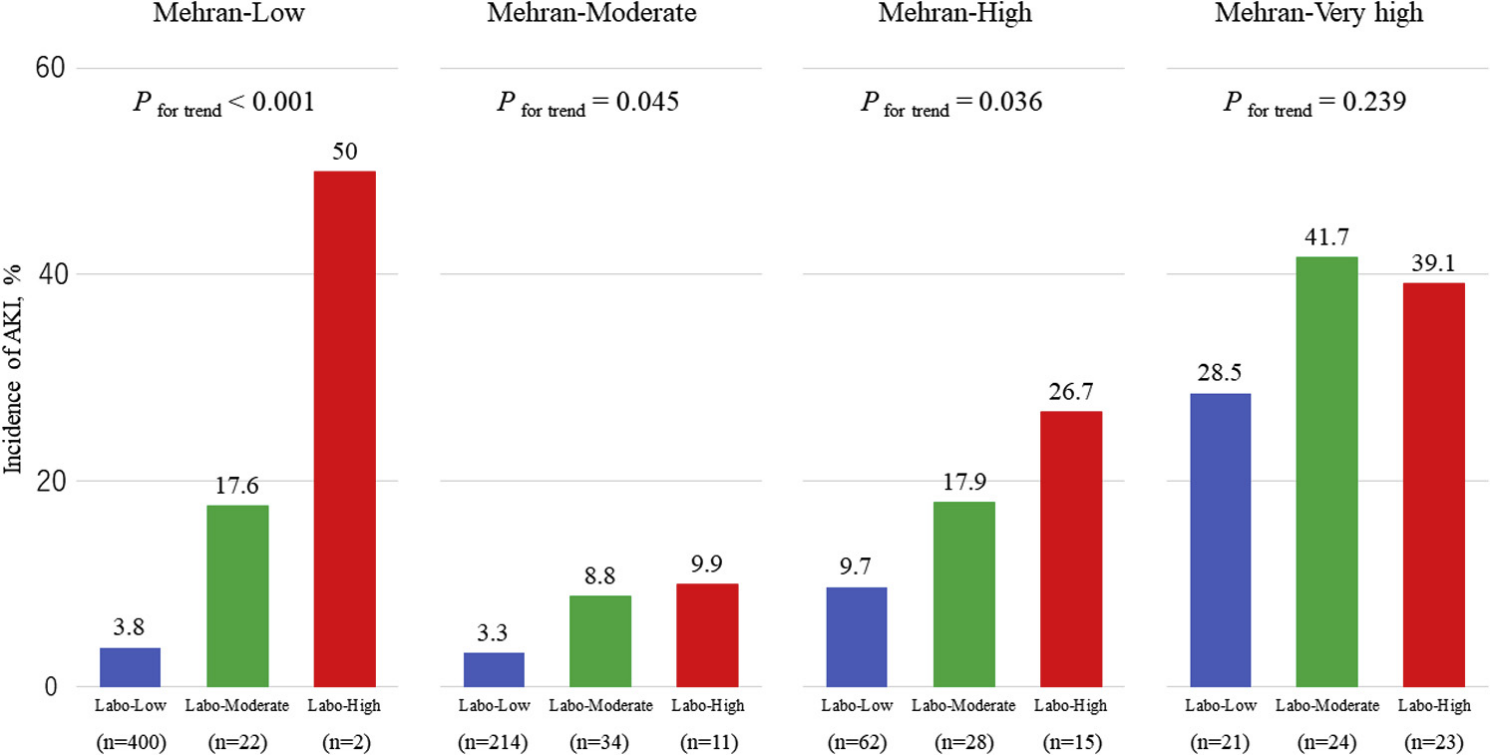
Reclassification of the Mehran-Based Risk for In-Hospital AKI by Applying the Laboratory-Based Risk Model Mehran-based risk was classified as follows: low (≤5), moderate (6-10), high (11 to 15), and very high (>15). Laboratory-based risk of the low-, moderate-, and high-risk subgroups was defined as 0 or 1, 2, and 3 or 4, respectively. AKI = acute kidney injury.

The AUC of the laboratory-based model and that applied to Mehran’s model in all cohorts was 0.755 (95% confidence interval: 0.696-0.805) and 0.749 (95% confidence interval: 0.681-0.807), respectively, and the predictive ability was similar between models (Figure 5). Furthermore, when patients in these 3 laboratory risk score subgroups were subdivided into Mehran’s 4 risk groups (low, moderate, high, very high) (11), our laboratory-based model could further stratify the risk of AKI in the low-, moderate-, and high-risk subgroups of Mehran’s model (all *P*_for trend_ <0.05) (Figure 6).

## Discussion

Key findings of the present study in Japanese patients with STEMI who underwent primary PCI were as follows: 1) each of 4 laboratory parameters quantified on admission (BS ≥200 mg/dL, hsTnI >1.6 ng/mL, albumin ≤3.5 mg/dL, and eGFR <45 mL/min/1.73 m^2^) was independently associated with an increased risk of AKI; 2) even though our model is composed of combined pre-procedural blood variables, it can be used easily and reliably to predict the risk of AKI; and 3) the predictive value of this model for the risk of AKI was comparable to that of the conventional Mehran model, and our model could further stratify that risk for the low-, moderate-, and high-risk subgroups identified by the Mehran model (Central Illustration). Thus, our findings suggest that this laboratory-based model is useful for the prediction of the post-procedural risk of AKI in patients with STEMI undergoing primary PCI.

**Central Illustration:**
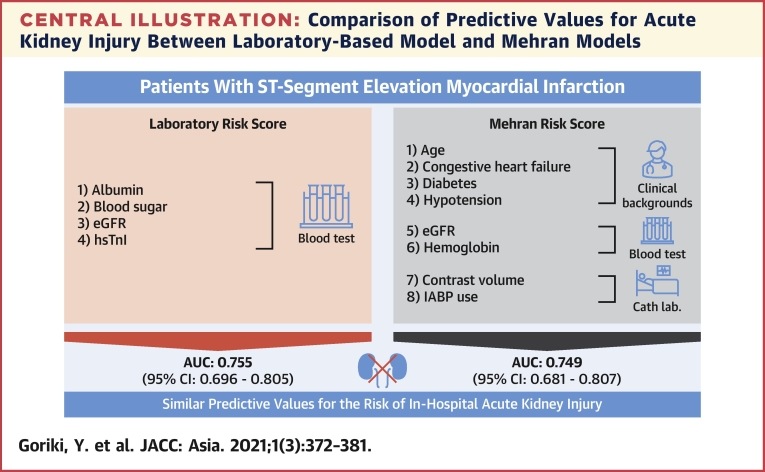
Comparison of Predictive Values for Acute Kidney Injury Between Laboratory-Based Model and Mehran Models We focused on routine pre-procedural blood tests to develop a risk score model for predicting in-hospital acute kidney injury in Japanese patients with ST-segment elevation myocardial infarction who underwent primary percutaneous coronary intervention. The predictive value of our laboratory-based model composed of 4 laboratory parameters was comparable to that of the conventional Mehran’s model composed of laboratory and nonlaboratory information. Our results suggest that this laboratory-based model is useful for early prediction of the post-procedural risk of acute kidney injury in that patient population. AUC = area under the curve; CI = confidence interval; eGFR = estimated glomerular filtration rate; hsTnI = high-sensitivity troponin I; IABP = intra-aortic balloon pumping.

AKI is the most common noncardiac complication in patients with STEMI and is strongly related to mortality after AMI (5,6). Hence, all patients with STEMI seen in the emergency department are required to receive a prompt risk assessment of AKI. Mehran’s model has been the most widely used risk score for predicting the risk of AKI. However, it has some clinical concerns that can make evaluation difficult, as follows. First, pre-procedural risk prediction is impossible, because Mehran’s model requires the dose of contrast material and use of mechanical support, such as IABP, which are not known before the procedure. Second, the hemodynamic state in patients with AMI, especially in emergency clinical settings, often fluctuates widely and rapidly and is modified by intravenous medications. Third, the patient’s clinical history is also needed for Mehran’s model, and that can be difficult to obtain from patients with clinical conditions, such as shock and cardiac arrest. In contrast, the results of blood tests can be obtained quickly and easily prior to the procedure, even in emergency department settings, and will provide completely objective information. We therefore focused on pre-procedural routine blood tests to develop a risk score model for predicting AKI in patients with STEMI who underwent primary PCI.

Few studies have assessed the combination of laboratory variables for AKI in patients with STEMI. Several biomarkers related to cardiac, metabolic, hematologic, and inflammatory responses have been reported to be independent predictors of AKI in patients with AMI (13,15, 16, 17, 18). Pre-existing chronic kidney disease is known as the strongest risk factor, with a lower level of kidney function associated with a higher degree of risk (11, 12, 13, 14). Our present study added the following 3 blood variables as potential predictors of AKI: hsTnI, albumin, and blood sugar.

Our study demonstrated a relationship between hsTnI and the risk of AKI in treated patients with STEMI. A previous report showed that troponin kinetics could be used to estimate the onset of ischemia in patients with STEMI, and this “biochemical ischemic time” correlates well with infarct size and long-term mortality (23). Previous AKI risk prediction models have focused on ischemia time or left ventricular ejection fraction (12,24). Thus, it is suggested that the incidence of AKI is associated with the degree of myocardial injury as assessed by hsTnI.

Inflammation and oxidative stress have been reported to be mechanisms underlying AKI (25). Acute coronary syndrome is an inflammatory state, and the serum albumin level often decreases in this situation (26). Therefore, the serum albumin level might have a role as an inflammatory marker in patients with STEMI. Moreover, albumin has antioxidant activities (27). In fact, several studies have suggested that albumin itself can protect the kidney from injury (28). Nonetheless, it is still unclear whether the unfavorable impact of hypoalbuminemia in the early phase of acute coronary syndrome reflects the state of inflammation or an independent effect of albumin itself.

Numerous studies have described an association between hyperglycemia on admission and AKI (16). The mechanisms underlying this relationship are explained by the fact that an acute increase in blood sugar suppresses flow-mediated vasodilatation, likely through increased production of oxygen-derived free radicals, and increases oxidative stress that may exacerbate the deleterious effects of contrast agents on the kidney (29,30). Moreover, acute hyperglycemia may induce a transient osmotic diuresis, resulting in volume depletion and dehydration, which are associated with an increased risk and severity of AKI.

Recently, Tsai et al (14). developed a new risk score model from data based on large numbers of patients undergoing PCI. That model was composed of 11 pre-procedural variables and showed good discrimination between STEMI patients with and without AKI. However, its computational estimation is somewhat complicated to perform in an emergency clinical setting. The patient’s clinical history is also needed for that model. Moreover, some AKI risk models have been reported in Asian populations (31,32). However, they are composed of variables similar to those in the conventional Mehran model (eg, vital signs, volume of contrast material, use of mechanical support, medical history). In contrast, our model is composed of variables that can be obtained easily before the procedure. Our model also showed adequate discrimination between patients with and without AKI that was comparable to that of the conventional Mehran model. Furthermore, our model could stratify each of the risk groups classified according to that model. Thus, our model has the potential to evaluate the risk of AKI earlier and more objectively than conventional models, even in emergency clinical settings.

### Study limitations

First, this was a single-center, retrospective, observational study of a relatively small sample. Second, this study evaluated laboratory parameters only upon admission for STEMI. Thus, it is difficult to determine if the parameters reflect acute pathophysiological changes associated with STEMI or if they are related to the conditions of the clinical backgrounds. Furthermore, the generalizability of the present findings to other patient populations, such as non-STEMI and other races, is also unknown. Third, we have no data on urine volume, although the Kidney Disease: Improving Global Outcomes guidelines advise defining AKI by urine volume as well (21). Therefore, this might lead to underestimation of the AKI rate. Finally, because we focused on routine pre-procedural blood tests to create the present prediction model, nonlaboratory clinical information (eg, age, comorbidities and medications), procedure-related factors (eg, contrast volume, use of IABP), and the severity of myocardial infarction (eg, cardiogenic shock, multivessel disease, Thrombolysis In Myocardial Infarction flow grades) were not accounted for. Therefore, those factors might be partly associated with the incidence of AKI in our study cohort. Nonetheless, our model showed a predictive performance comparable to that of Mehran’s model. Of note, a previous study showed that amount of contrast media, which is incorporated into Mehran’s model, was not associated with development of AKI in patients with STEMI (4). Further research would be needed to assess whether the other clinical features could increase the predictive value for the incidence of AKI in patients with STEMI.

## Conclusions

Our novel laboratory-based model might be helpful for early prediction of the risk of AKI in patients with STEMI who undergo primary PCI within 48 hours after onset.

Perspectives **COMPETENCY IN MEDICAL KNOWLEDGE:** This laboratory-based model might be helpful for early prediction of the risk of in-hospital AKI in patients with STEMI who undergo primary PCI. Furthermore, its overall accuracy is comparable to that of the conventional Mehran contrast-induced nephropathy risk score model, and our model could further stratify risk across the risk status categories of the Mehran model.

**TRANSLATIONAL OUTLOOK:** Prospective clinical trials are needed to validate the predictive accuracy of our laboratory model.

## Funding Support and Author Disclosures

This work was supported by Japan Society for the Promotion of Science KAKENHI Grant Number JP21K08130. The authors have reported that they have no relationships relevant to the contents of this paper to disclose.
