# Supplementary material for: A Novel Prediction Model of Acute Kidney Injury Based on Combined Blood Variables in STEMI
Source: JACC Asia. 2021 Oct 26;1(3):372–81. doi: 10.1016/j.jacasi.2021.07.013 (PMC9627908; doi:10.1016/j.jacasi.2021.07.013)
Supplement: Supplemental Tables 1 and 2 [file mmc1.docx]

**Supplemental Table 1.** Univariate Variate Analysis of Variables for In-Hospital AKI in the Randomized Derivation Cohort.

|  | **Non-AKI** | **AKI** | **P value** |
| --- | --- | --- | --- |
| WBC, ×10^2^ /μL | 104.0 ± 36.7 | 114.3 ± 43.4 | 0.041 |
| Hemoglobin, g/dL | 14.1 ± 2.0 | 13.4 ± 2.5 | 0.027 |
| Platelet, ×10^4^/μL | 22.4 ± 6.0 | 20.2 ± 6.4 | 0.009 |
| HbA1c, % | 6.0 [5.7, 6.5] | 6.2 [5.7, 7.3] | 0.141 |
| BS, mg/dL | 156 [132, 198] | 204 [155, 282] | < 0.001 |
| eGFR, mL/min/1.73m^2^ | 68.7 ± 21.5 | 56.1 ± 26.3 | < 0.001 |
| LDL-CHO, mg/dL | 126.8 ± 34.5 | 120.8 ± 44.7 | 0.220 |
| HDL-CHO, mg/dL | 47.1 ± 12.2 | 44.1 ± 12.3 | 0.070 |
| Albumin, mg/dL | 4.1 ± 0.5 | 3.7 ± 0.6 | < 0.001 |
| Uric acid, mg/dL | 5.9 ± 1.5 | 6.4 ± 1.7 | 0.010 |
| CRP, mg/dL | 0.12 [0.06, 0.34] | 0.23 [0.07, 0.37] | 0.021 |
| CK, IU/L | 153 [93, 389] | 232 [98, 1274] | 0.016 |
| hsTnI, ng/mL | 0.20 [0.03, 2.02] | 1.33 [0.18, 29.1] | < 0.001 |
| BNP, pg/mL | 42.8 [17.2, 123.8] | 148.3 [45.2, 437.5] | < 0.001 |

Data for continuous variables are given as mean ± standard deviation for normal distribution or median [interquartile range] for skewed distribution.

AKI, acute kidney injury; BNP, brain natriuretic peptide; BS, blood sugar; CK, creatine kinase; CRP, C-reactive protein; eGFR, estimated glomerular filtration rate; HDL-CHO, high-density lipoprotein cholesterol; hsTnI, high-sensitive troponin I; LDL-CHO, low-density lipoprotein cholesterol; WBC, white blood cell.

**Supplemental Table 2.** Multivariate Logistic Regression Analysis in the Randomized Derivation Cohort and Corresponding Risk Score for AKI.

|  | **Odds ratio** | **95% confidence interval** | **P value** | **Score** |
| --- | --- | --- | --- | --- |
| BS ≥200 mg/dL | 2.83 | 1.59 – 5.04 | 0.004 | 1 |
| hsTnI >1.6 ng/dL (normal upper limit×50) | 2.13 | 1.20 – 3.80 | 0.010 | 1 |
| Albumin ≤3.5 mg/dL | 2.88 | 1.50 – 5.50 | 0.001 | 1 |
| eGFR <45 mL/min/1.73 m^2^ | 2.46 | 1.31 – 4.64 | 0.005 | 1 |

AKI, acute kidney injury; BS, blood sugar; eGFR, estimated glomerular filtration rate; hsTnI, high-sensitive troponin I..
